# Combination of Vildagliptin and Ischemic Postconditioning in Diabetic Hearts as a Working Strategy to Reduce Myocardial Reperfusion Injury by Restoring Mitochondrial Function and Autophagic Activity

**DOI:** 10.15171/apb.2018.037

**Published:** 2018-06-19

**Authors:** Goltaj Bayrami, Alireza Alihemmati, Pouran Karimi, Aniseh Javadi, Rana Keyhanmanesh, Mustafa Mohammadi, Milad Zadi-Heydarabad, Reza Badalzadeh

**Affiliations:** ^1^Drug Applied Research Center, Tabriz University of Medical Sciences, Tabriz, Iran.; ^2^Neurosciences Research Center, Tabriz University of Medical Sciences, Tabriz, Iran.; ^3^Cardiovascular Research Center, Tabriz University of Medical Sciences, Tabriz, Iran.; ^4^Student Research Committee, Tabriz University of Medical Sciences, Tabriz, Iran.; ^5^Department of Physiology, Faculty of Medicine, Tabriz University of Medical Sciences, Tabriz, Iran.; ^6^Molecular Medicine Research Center, Tabriz University of Medical Sciences, Tabriz, Iran.

**Keywords:** Ischemic postconditioning, Diabetes, Reperfusion injury, Autophagy, Mitochondrial function

## Abstract

***Purpose:*** Diabetic hearts are resistant to cardioprotection by ischemic-postconditioning (IPostC). Protection of diabetic hearts and finding related interfering mechanisms would have clinical benefits. This study investigated the combination effects of vildagliptin (Vilda) and IPostC on cardioprotection and the levels of autophagy and mitochondrial function following myocardial ischemia/reperfusion (I/R) injury in type-II diabetic rats.

***Methods:*** Diabetes was established by high fat diet/low dose of streptozotocin and lasted for 12 weeks. The diabetic rats received Vilda (6 mg/kg/day, orally) for one month before I/R. Myocardial regional ischemia was induced through the ligation of left coronary artery, and IPostC was applied immediately at the onset of reperfusion. The infarct size was assessed by a computerised planimetry and left ventricles samples were harvested for cardiac mitochondrial function studies (ROS production, membrane potential and staining) and western blotting was used for determination of autophagy markers.

***Results:*** None of Vilda or IPostC but combination of them could significantly reduce the infarct size of diabetic hearts, comparing to control (P<0.001). IPostC could not significantly affect p62 expression level in diabetic hearts, but pre-treatment with Vilda alone (p<0.05) and in combination with IPostC (p<0.01) more significantly decreased p62 expression in comparison with corresponding control group. The expression of LC3B-II and LC3BII/LC3BI as well as mitochondrial ROS production were decreased significantly in treatment groups (p<0.001). Mitochondrial membrane depolarization was significantly higher and mitochondrial density was lower in untreated diabetic I/R hearts than treated groups (p<0.001). IPostC in combination with vildagliptin prevented the mitochondrial membrane depolarization and increased the mitochondrial content more potent than IPostC alone in diabetic hearts.

***Conclusion:*** Combination of vildagliptin and IPostC in diabetic hearts was a well-working strategy to reduce myocardial I/R damages by restoring mitochondrial membrane potential and ROS production and modulating the autophagic activity in I/R hearts.

## Introduction

Myocardial ischemia/repefusion (I/R) injury is defined as the reversible or irreversible cardiomyocyte damages following reperfusion therapy, leading to excessive reactive oxygen species (ROS) generation, inflammatory reactions, mitochondrial dysfunction and subsequently cell death.^1^ Diabetes mellitus is a main cardiovascular comorbidity which exacerbates myocardial function following myocardial I/R injuries and reduces the efficiency of cardioprotective interventions.^2,3^ Diabetic heart is accompanied with the development of cardiac dysfunction, myocardial hypertrophy, myocardial interstitial fibrosis, increased apoptosis, mitochondrial dysfunction, dysregulated autophagy and upregulation of oxidative stress and pro-inflammatory mediators.^4,5^ The prevalence of type-II diabetes is rapidly increasing world-wide and insulin resistance is a critical metabolic abnormality among these diabetic patients, resulting in hyperglycemia, hyperinsulinemia, pancreatic β-islet cell dysfunction in advanced stages of diabetes and lack of insulin effects in the periphery.^4,6^

Autophagy is a bulk degradation of long-live protein, oxidized proteins and dysfunctional organelles inside the cells. Both protective or negative roles of autophagy have been reported in experimental settings of I/R injury and diabetes, depending on the type and duration of the stress conditions in different organs.^7,8^ Thus, whether the alternations in autophagy are beneficial or detrimental under I/R injury remains controversial and needs more investigations, particularly in diabetes. It is evident that modification of the excessive autophagic response via various means would be associated with the restoration of myocardial functions during I/R insults.

Ischemic postconditioning (IPostC) is a clinically applicable cardioprotective intervention in which some short alternative cycles of ischemia and reperfusion is applied immediately at the early minutes of reperfusion.^5,9^ It is a mechanical strategy with significant cardioprotection in non-diabetic (healthy) animal experiments. Recent evidence showed that autophagy can be regulated by postconditioning during reperfusion.^9,10^ In addition, numerous studies showed that the diabetes abrogates cardioprotective effects of IPostC following myocardial reperfusion injury, due to the numerous less-known underlying mechanisms, including dysfunctions in cell survival signaling pathways.^11,12^ Using an adjuvant therapy to increase the potency of IPostC in diabetic myocardium may be one of the promising strategies to restore the diabetes-induced loss of cardioprotection.^13^

Vildagliptin (as a potent dipeptidyl peptidase 4 (DPP-4) inhibitor) acts as an enhancer of glucagon-like peptide 1 (GLP-1) hormone, having multiple beneficial effects besides reducing hyperglycaemia, such as increasing glucose-stimulated insulin release,^14^ improving mitochondrial function,^15^ and anti-inflammatory^16^ and antioxidative^17^ effects. Also, it has been reported to exert positive cardiovascular effects in both preclinical and clinical studies under healthy or diabetic circumstances.^15,18^ In our previous work, we showed that pre-treatment of diabetic rats with vildagliptin could improve the failure of IPostC on the recovery of myocardial hemodynamic function after ischemic event and this effect was associated somewhat with anti-oxidative and anti-inflammatory properties.^19^ There is less or no information about the effect of vildagliptin on autophagy and mitochondrial function in normal condition or in type-II diabetic myocardium following I/R injury.^20,21^ Due to the positive therapeutic potentials of vildagliptin in cardiovascular medicine, in this experiment we have combined vildagliptin and IPostC to investigate their cardioprotective effects by focusing on autophagy and mitochondrial function in (chronic) type-II diabetic hearts with regional I/R injury.

## Materials and Methods

### Animals

The 8-week-old male Wistar rats (200-250g) were used in this study. The rats were housed in animal room with free access to food and water and maintained at 22±2°C on a 12 h light/dark cycle. They were under strict surveillance until the end of the study.‏ All steps of the experiment were carried out in line with the Guide for the Care and Use of Laboratory Animals published by the US National Institutes of Health (NIH publication No 85-23, revised 1996) and officially accepted by the Ethical Commission of Tabriz University of Medical Sciences.

#### Induction of type-II diabetes

Type-II diabetes with insulin resistance was induced by the method of high*-*fat diet-fed and low*-*dose streptozotocin, according to the previous reports.^22^ The diabetic period was lasted for 12 weeks for simulation of chronic diabetes. After a week of acclimatization, diabetic rats were fed for 6 weeks *ad libitum* with a high-fat diet, containing 35% normal pellet, 30% lard, 4% sucrose, 24% casein, 1% cholesterol, 0.3% DL-Methionine (62% calories from fat, and total caloric value was about 4.6 kcal/g). Non-diabetic healthy rats were fed with normal diet. At the end of 6th week, the diabetic rats were then administered with a low dose of streptozotocin (35 mg/kg in citrate buffer, pH 4, i.p.) after 8 hours fasting. Blood samples were obtained 72 hours after STZ injection for confirmation of diabetes. Rats with blood glucose levels higher than 250 mg/dl were enrolled to the further study.

#### Experimental Preparation and Protocols

The animals were randomly divided into five diabetic and two non-diabetic subgroups, n=5-6/each. Diabetic (D) groups included: D-Sham, D-Control, D-IPostC (diabetic hearts of rats received IPostC), D-Vilda (diabetic rats received vildagiliptin) and D-Vilda-IPostC (diabetic rats received vildagiliptin and then their hearts received IPostC). Non-diabetic healthy (H) animals were divided into H-Sham and H-Control subgroups. Rats in D-Vilda and D-Vilda-IPostC groups were treated with vildagliptin (6 mg/kg/day) through feeding gavage in the last 5 weeks.^15,19^

#### Myocardial I/R modeling

At the end of treatment, all hearts of animals were isolated as mentioned previously.^23,24^ Briefly, before the anesthetization of rats with the intra-peritoneal injection of ketamine (60 mg/kg) and xylasine (10 mg/kg), they were heparinized (500 IU) to avoid blood clotting during surgery. Then, the hearts of animals were rapidly isolated and mounted on a constant-pressure (80 mmHg) mode of Langendorff perfusion apparatus (ML176-V, AD Instruments, Australia) and perfused with a Krebs–Henseleit solution containing (in mM/l): NaCl 118; KCl 4.7; CaCl_2_ 2.5; MgSO_4_ 1.2; NaHCO_3_ 25; KH_2_PO_4_ 1.2; glucose 11.1. The perfusion solution was gassed with a mixture of 95% O2, 5% CO2 at 37°C and pH 7.4. For measurement of interventricular pressure changes, a water-filled latex balloon was inserted into the left ventricle and the signals were delivered to the related transducer through a connecting pressure catheter. In all experimental groups, a 5-10 mmHg of left ventricular end-diastolic pressure was produced by adjusting the balloon volume. Following stabilization on Langendorff perfusion system, the isolated hearts were assigned to receive one of these protocols:

- H-Sham: the hearts were subjected to 105 min full perfusion only.
- D-Sham: the hearts were subjected to 105 min full perfusion only.
- H-Control: the hearts were subjected to 35 min ischemia + 60 min reperfusion.
- D-Control: the hearts were subjected to 35 min ischemia + 60 min reperfusion.
- D-IPostC: the hearts were subjected to 35 min ischemia + 6 cycles of 10s ischemia /10s reperfusion immediately at the onset of 60 min reperfusion.^25^
- D-Vilda: the hearts of vildagliptin-receiving rats were subjected to 35 min ischemia + 60 min reperfusion
- D-Vilda-IPostC: the hearts of vildagliptin-receiving rats were subjected to 35 min ischemia + 6 cycles of 10s ischemia /10s reperfusion immediately at the onset of 60 min reperfusion.

Regional ischemia (for 35 minutes) was induced by tightening a 5-0 silk suture around the left anterior descending (LAD) coronary artery, close to its origin. The snare was then removed and the hearts received reperfusion for 60 minutes. At the end of reperfusion, the left ventricle of each heart was isolated from whole heart and then the ischemic zone were harvested and divided in half, one part were paraffinized for mitochondrial staining method and the other part frozen in liquid nitrogen and stored at -70°C until use.

#### HOMA1-IR index measurement for confirmation of type-II diabetes and insulin resistance 

Fasting blood glucose level was measured with a glucometer device (elegance CT-X12, Convergent Technologies, Germany) and fasting plasma insulin levels were determined using a rat-specific insulin ELISA kit (Cayman chem., Ann Arbor, MI, USA) according to the manufacturer’s instructions. Insulin resistance was identified with the homeostasis model assessment of insulin resistance (HOMA1-IR) index, using the following formula: fasting blood glucose (mmol/l) × fasting insulin (µu/l)/22.5.^26^

#### Infarct size evaluation

In a separate grouping of animals, the infarct sizes were measured as described previously.^23^ At the end of reperfusion, the LAD coronary artery was occluded again and the hearts were perfused with 2 ml Evans blue dye (0.25%). Then, 2 mm slices were prepared from the hearts and immersed in 2,3,5-triphenylte-trazolium chloride (TTC, 1%) in phosphate buffer solution (pH 7.4). A computerized planimetry and ImageJ software were employed to determine the Left ventricular, risk zones and infarcted areas, by an investigator blinded to the animal grouping. The infarct sizes were reported as a percentage of the left ventricular risk zones.

#### Preparation of tissue homogenates

At the end of each Langendorff experiment, the left ventricles were separated and the ischemic zones were sampled and cut into pieces in lysis buffer containing (mM/ml) 1 KH2PO4, 1 KCL, 50 Tris–HCl pH 7.4, 1 EDTA, 1 NaF, 1 Na3VO4, and 1% triton 100X and protease inhibitor cocktail (Sigma-Aldrich, USA) and then homogenized with a Dounce glass tissue homogenizer (Sigma, Germany) in ice-cold. The homogenate was underwent centrifugation at 10,000 RCF for 10 min at 4°C. The obtained supernatants were quickly frozen at -70°C.^23^ The Bradford method was used for detection of the amount of proteins in supernatants.^27^

#### Western blotting

The western blotting was performed as described previously.^20^ Briefly, protein of the samples were separated in 12% SDS-gels, and then transferred to PVDF membranes at an hour for all gels. Subsequently, the blocking of membranes were done in 5% skim milk buffer containing 0.1% Tween-20 for 1.5 h and then probed with primary antibodies against microtubule-associated proteins 1A/1B light chain 3B (LC3B) (1:1000, Cell Signaling), P62/SQSTM1 (1:500, Santa Cruz) and β-actin (1:500, Cell Signaling) overnight at 4°C on a shaker incubator. After 4×5 min washing with Tris buffer saline containing 0.1% Tween-20, HRP-conjugated secondary antibody (1:7000, Cell Signaling) was added on the membranes. After for an hour incubation on shaker, the membranes were bathed in wash buffer and washed at least 3×5 min. Then, the membranes were incubated with the enhanced chemiluminescence (ECL, Amersham) reagents in dark room. This step was followed by the exposing of the membrane to an X-ray film and visualization of the chemiluminescence of the binding by means of a visualizing machine. The intensity of the bands was calculated using Image J software (IJ 1.46r version, NIH, USA) and normalized to each sample based on the intensity of β-actin as internal control.

### Mitochondrial function studies

#### Isolation of cardiac mitochondria

For mitochondrial studies, at the end of reperfusion time of each isolated heart, the area at risk (AAR) of left ventricle was rapidly harvested for mitochondria isolation. All operations were performed on ice at 4°C, freshly. Myocardial AAR (40 mg) was minced with a scissor and homogenized in isolation buffer containing 70 mmol/L sucrose, 210 mmol/L mannitol, and 1 mmol/L EDTA in 50 mmol/L Tris/HCl, pH 7.4 (approx.1 mL buffer/15 mg tissue) by pre-cooled 2.0-ml Dounce Homogenizer. After centrifugation of the homogenates at 1300*g* for 3 minutes, the supernatant was centrifuged again at 10000*g* for 10 minutes. The mitochondrial pellet was obtained and suspended in 100 µl storage buffer containing 70 mmol/L sucrose and 210 mmol/L mannitol in 50 mmol/L Tris/HCl, pH 7.4 and was used freshly within 4 hours. Protein content was assayed by the bicinchoninic acid method with BSA as a standard.^28^

#### Cardiac mitochondrial ROS production

For mitochondrial ROS production study, the mitochondrial pellets were incubated with 2 µM dichlorofluorescin diacetate (DCFDA) dye at room temperature for 30 minutes. DCFDA, a fluorogenic dye, measures hydroxyl, peroxyl and other ROS activity within the cell and its organelles. After diffusion into organelle, DCFDA is oxidized by ROS into a highly fluorescent compound, 2', 7'-dichlorofluorescein (DCF). DCF fluorescence was detected by a fluorometric method with the excitation and emission wavelengths of 480 nm and 530 nm, respectively. An increase in the fluorescent intensity indicated an increased mitochondrial ROS production.^29^

#### Cardiac mitochondrial membrane potential changes

Cardiac mitochondrial membrane potential changes were determined using the 5,5',6,6'-tetrachloro-1,1',3,3'-tetraethylbenzimidazolylcarbocyanine iodide (JC-1) by an isolated mitochondrial staining kit according to the manufacturer’s protocol (Sigma, Germany). In brief, 5 μg isolated mitochondria were diluted in 100 μl assay buffer containing 0.2 μg/ml JC-1 and incubated in the dark at room temperature for 10-20 min. JC-1 aggregates (red fluorescent, in healthy cells) were exited at 525 nm wavelength and detected at 590 nm emission wavelength, and JC-1 monomers (green fluorescent, in unhealthy cells) were exited at 485 nm wavelength and detected at 530 nm emission wavelength. Fluorescent intensity was recorded using spectrofluorometer (FP-750). A decrease in the red/green fluorescent intensity ratio is a representative of depolarization of mitochondrial membrane.^30^

#### Cain's method for staining mitochondria 

The ischemic zone of left ventricular sections from mid-myocardial parts were deparaffinized and placed in hot aniline acid-fuschin solution for about 10 min and incubated in 0.1% sodium carbonate solution until pale pink. Following a quick rinse in 1% hydrochloric acid, sections were rinsed in distilled water, and then counterstained in methyl blue. Sections were again rinsed in water, dipped in 1% HCl, rinsed once more in water, and then dehydrated in graded alcohols and cleared in Xylol and infiltrated in paraffin. Sections were imaged using Olympus Bx40 microscope (Olympus Japan),^31^ then evaluated according to Table 1. All processes of histological evaluations were done in a blind and random manner.

### Statistical analysis

All values were expressed as means ± SEM. Comparison between groups were analysed through *one-way ANOVA* followed by *Tukey* post hoc or Student’s t-tests as appropriate, using SPSS v16. A p<0.05 was considered statistically significant.

**Table 1:** Grading system for evaluation of mitochondrial density in cardiac cells.

**Scoring**	**Intensity of Staining**	**Percentage Score**
**1**	weak staining	< 25% positive
**2**	moderate staining	25% - 50% positive
**3**	strong staining	> 50% positive

## Results

### FBS **and** HOMA1-IR index analysis

Analysis of variance showed that the diabetic rats had a significantly higher blood glucose, plasma insulin and HOMA1-IR index (p<0.001, for all) comparing to those of non-diabetic control group. Pre-treatment of diabetic rats with vildagliptin significantly reduced hyperglycaemia (p<0.001) and increased plasma insulin level (p<0.001) and decreased HOMA1-IR index (p<0.05) comparing to the diabetic group (Table 2).

**Table 2:** Fasting blood sugar (FBS), plasma level of insulin and HOMA-IR test at the end of treatment protocol.

**Groups**	**Non-Diabetic**	**Diabetic**	**Diabetic+Vilda**
**FBS (mmol/l)**	5.28±0.18	28.60±0.50^***^	20.06±0.25^***^
**Insulin (µu/ml)**	4.64± 0.27	7.33±0.14^***^	9.52±0.20^***^
**HOMA1-IR**	1.08±0.05	9.31±0.18^***^	8.23±0.50^***^

The data were expressed as mean ± SEM. n=10 for each group. Vilda: vildagliptin; HOMA1-IR: homeostasis model assessment-estimated insulin resistance.^***^p<0.001 as compared with non-diabetic group and ^++^p<0.01 and ^+++^p<0.001 as compared with diabetic group.

### Myocardial Infarct size

**Figure 1:**
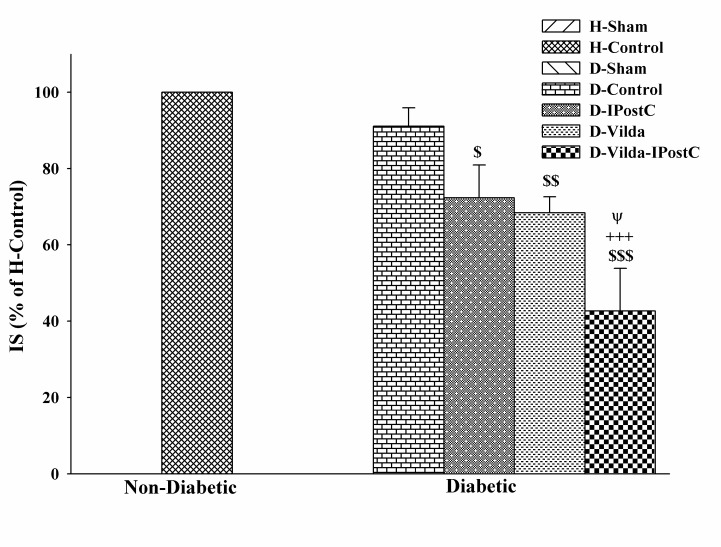
**The changes of infarct sizes (IS) in experimental groups.** IS was evaluated by evan's blue and triphenyltetrazolium chloride staining method, in non-diabetic and diabetic hearts subjected to 35 min regional ischemia and 60 min reperfusion. The data were expressed as mean±SEM. n=5-6 for each group. H: healthy; D: diabetic; IPostC: ischemic postconditioning; Vilda: vildagliptin. ^$^p<0.05, ^$^p<0.01 and ^$^p<0.001 as compared to H-control;^+++^p<0.001 as compared to D-Control group; ^Ψ^p<0.05 as compared to D-Vilda and D-IPostC groups.

Induction of I/R injury through occluding of LAD provided similar risk zones in both control and diabetic hearts and this indicates no difference in the degree of ischemic stimulus in all groups. Instead, I/R injury led to induction of 42±2% infarct size in control hearts vs. 38±2% in diabetic hearts. Neither IPostC nor vildagliptin significantly reduced the infarct size in diabetic rats (17.02% and 21.73% lower than those of D-Control group, respectively). However, combined administration of IPostC with vildagliptin had a greater and more significant infarct sparing effect (up to 48.18% reduction) as compared with diabetic control group (P<0.001) (Figure 1).

### Detection of autophagy and autophagic flux

**Figure 2:**
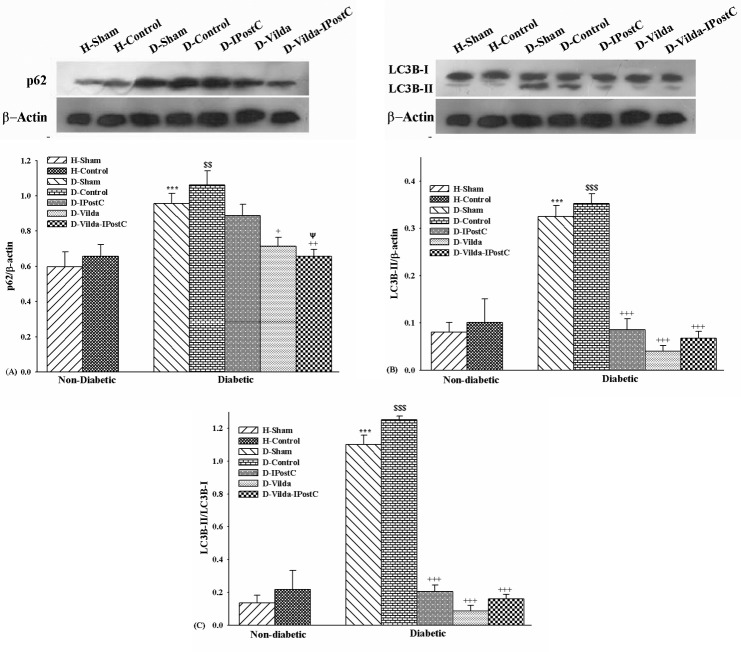
**Changes of autophagy markers in experimental groups.** The data were expressed as mean ±SEM. n=5 for each group. **A)** Changes of p62 protein expression; **B)** Changes of LC3B-II protein expression; and **C)** Changes of the ratio of LC3BII/LC3BI. H: healthy; D: diabetic; IPostC: ischemic-postconditioning; Vilda: vildagliptin. Levels of β-actin protein were used as the loading control.^$$^p<0.01 and ^$$$^p<0.001 as compared with H-Control group;^+^p<0.05, ^++^p<0.01 and ^+++^p<0.001 as compared with D-Control group; ^ΨΨ^p<0.001 as compared with D-IPostC group.

To determine the autophagic changes, western blotting were performed for the autophagic markers, proteins LC3B-II and p62 (a selective substrate of autophagy) in area at risk of left ventricle in all groups. LC3B-II expression correlates with the increased levels of autophagic vesicles. P62 is subjected to the autophagic degradation at the lysosome, so autophagic flux is indicated by a reduction in the level of this proteins. The results revealed that in H-Control group the expression of p62 as well as expression of LC3BII and the ratio of LC3BII/LC3BI were increased to some extent as compared with those of H-Sham group, but there was no significant difference between them (Figure 2-A and B). It seems that autophagy tends to be unregulated very slightly with I/R injury in healthy hearts. In diabetic hearts, the expression of p62 and LC3B-II and the ratio were significantly increased in D-Sham group as compared with H-Sham healthy group (p<0.001) (Figure 2-A and B). These results showed that in this chronic model of type-II diabetes after 12 weeks, the autophagy was unregulated but the autophagic flux was more likely impaired at the late process of lysosomal degradation, as indicated indirectly by the lack of reduction of p62 level in diabetic hearts.

As shown in Figure 2, autophagic activity in D-Control group was significantly increased as compared with D-Sham group (p<0.01 for p62, and p<0.001 for both LC3B-II and the ratio).

Treatment of diabetic hearts decreased the expression of p62, so that this reduction was statistically significant in groups D-Vilda (p<0.05) and D-Vilda-IPostC (p<0.01) in comparison with D-Control group (Figure 2-A). IPostC alone could not significantly influence the levels of p62 expression in diabetic hearts, but the combination of IPostC with vildagliptin pre-treatment modulated the p62 levels significantly as compared with IPostC group (p < 0.05). In addition, the expression of LC3B-II and the ratio of LC3BII/LC3BI were decreased significantly in all treatments groups as compared with D-Control group (p<0.001 for all). While, the expression of LC3B-I was similar among all groups. These results showed that in treated diabetic hearts subjected to I/R injury, autophagy were decreased as compared with diabetic I/R heart with no treatments. It seems that addition of vildagliptin to IPostC administration increased the potency of IPostC to reduce the expressions of p62 and LC3-II proteins expression, and vildagilptin restored the autophagy flux in diabetic I/R hearts receiving IPostC.

### Mitochondrial function

#### Cardiac mitochondrial ROS production

**Figure 3:**
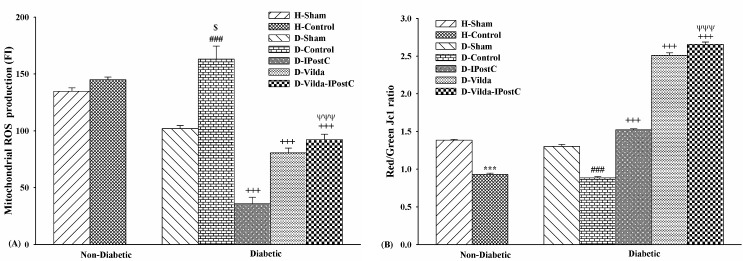
**Mitochondrial functional studies. A)** Cardiac mitochondrial ROS production assessed by DCFH-DA dye; and** B)** Cardiac mitochondrial membrane potential changes assessed by JC-1 dye. The data were expressed as mean ± SEM. n=6 for each group. H: Healthy; D: diabetic; IPostC: ischemic postconditioning; Vilda: vildagliptin. ^***^p<0.001 as compared with H-Sham group; ^###^p<0.001 as compared with D-Sham group; ^$^p<0.05 as compared with H-Control group; ^+++^p<0.001 as compared with D-Control group; ^ΨΨΨ^p<0.001 as compared with D-IPostC group.

The level of cardiac mitochondrial ROS production in isolated mitochondria from area at risk of myocardium was expressed as fluorescence intensity of DCF, indicating the levels of ROS production. Figure 3-A showed that the levels of ROS production between groups were statistically significant (p<0.001). ROS production level in control hearts was higher than sham hearts, but this was statistically significant only in D-Contol group compared to D-Sham group (p<0.001). ROS production levels were significantly reduced in D-IpostC, D-Vilda and D-Vilda-IPostC groups in comparison with those of D-Control group (p<0.001, for all). In addition, the level of ROS production in vildagliptin-treated diabetic hearts was higher than D-IPostC hearts (Figure 3-A).

#### Cardiac mitochondrial membrane potential changes

Changes of cardiac mitochondrial membrane potential were expressed as red/green fluorescent intensity ratio of JC-1 staining in isolated mitochondria from area at risk of the myocardium (Figure 3-B). The greater red/green ratios correlate with normal membrane potentials (lack of unwanted depolarization). The ratio was significantly lower in control groups versus sham groups in non-diabetic and diabetic hearts (p<0.001/both), indicating the decreased or collapsed membrane potentials (more depolarization) in these I/R hearts. Mitochondrial membrane depolarization was significantly higher in D-Control than other treated-diabetic groups (p<0.001); thus, IPostC alone, vildagliptin alone and combination of vildagliptin with IPostC prevented the further depolarization of mitochondrial membrane potential. In addition, the ratio of red/green fluorescent intensity in combination treatment was significantly higher than those of IPostC alone group (p<0.001), indicating the increased efficiency of IPostC on mitochondrial functional preservation, when combined with vildagliptin pre-treatment (Figure 3-B).

### Mitochondrial density

**Figure 4:**
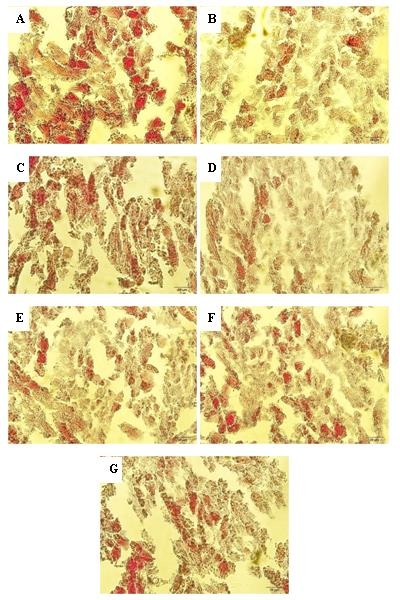
**Photomicrographs of mitochondrial density in cardiomyocytes of experimental groups.** Area at risk of left ventricle sections stained using Cain's method for mitochondrial density. Scale bar: 20 µm. A) H-Sham; B) H-Control; C) D-Sham; D) D-Control; E) D-IPostC; F) D-Vilda; G) D-Vilda-IPostC groups.

**Figure 5:**
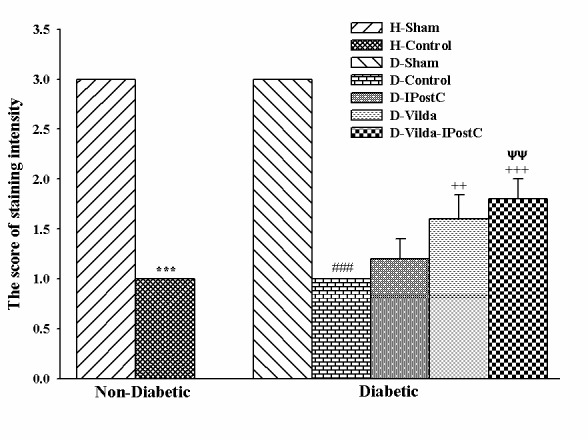
**The scoring of staining intensity for determining mitochondrial densities by Cain's method.** The data were expressed as mean ± SEM. n=5 for each group. H: Healthy; D: diabetic; IPostC: ischemic postconditioning; Vilda: vildagliptin. ^***^p<0.001 as compared with H-Sham group; ^###^p<0.001 as compared with D-Sham group; ^++^ p<0.001, ^+++^p<0.001 as compared with D-Control group; ^ΨΨ^p<0.01 as compared with D-IPostC group.

To test whether mitochondrial content and density was affected in area at risk of left ventricle, the myocardial sections from all groups were subjected to histological analysis using Cain's Method to specifically visualize the mitochondria. Increased intensity of staining indicates to increased mitochondrial density in Cain’s method. As indicated by histological images in Figure 4 and intensity scoring in Figure 5, the mitochondrial density was significantly lowered in H-Control group versus H-Sham group and so in D-Control group versus D-Sham group (p<0.001 for both). In addition, mitochondrial density was significantly increased in vildagliptin-treated diabetic groups as compared with D-Control group (p<0.01, and p<0.001, respectively). However, higher mitochondrial densities were seen in hearts treated with combination of vildagliptin and IPostC compared to IPostC and D-Control hearts (p<0.01) (Figure 5).

## Discussion

This experimental study showed that pretreatment of type-II diabetic rats with vildagliptin significantly restored the positive effects of IPostC on cardioprotection, autophagic flux and mitochondrial function following reperfusion injury, characterized by improved myocardial infarction, decreased autophagic markers LC3B-II and p62, reduced mitochondrial ROS generation, decreased mitochondrial membrane depolarization and increased mitochondrial number and density. Type-II diabetic patients with insulin resistance are characterized by hyperglycemia, hyperlipidemia, hyperinsulinemia, glucose intolerance and insulin resistance.^6^ In this study, the diabetic rats demonstrated glucose intolerance at the end of 7^th^ week and high FBS, hyperinsulinemia and increased HOMA1-IR at the end of 12^th^ week, confirming that the animals were suffered with type-II diabetes and insulin-resistance. Oxidative stress, impaired mitochondrial function, apoptotic and autophagic stresses are common in the pathophysiology of diseases like diabetes and I/R injury.^4,32^

In type-II diabetes, increased plasma lipid profile leads to increased free fatty acid (FA) levels, because of inability of insulin to suppress the hormone sensitive lipase in the adipose tissue and the secretion of very low-density lipoproteins in the liver. In this condition, myocardial metabolism is switched to FA uptake and oxidation.^33^ Therefore, diabetic heart is subjected to excessive oxidative stress, inflammation and lipotoxiciy that contribute to insulin resistance, functional impairment of the mitochondria, cardiomyocyte hypertrophy, apoptosis and dysregulation of autophagic activity.^34^ Vildagilptin, as an enhancer of GLP1 hormone, increased plasma levels of insulin, decreased glucose intolerance and HOMA1-IR, so increased insulin sensitivity in our type-II diabetic rats. Some parts of these effects of vildagliptin can come back to its improving effects on the diabetes-induced alterations in plasma lipid profile.^19^ At the time of reperfusion in diabetic hearts, various intracellular alterations switch the source of energy requirements to more fatty acid metabolism, inducing rapid opening of mitochondrial permeability transition pore (mPTPs) and decreasing mitochondrial membrane potential. These changes, in turn, cause overproduction of ROS by dysfunctional mitochondria, thus a defective cycle develops and leads to irreversible cellular injury triggered by necrosis, apoptosis and autophagy process.^1,35,36^

At basal level, autophagy has physiological function in the heart by restoring normal energy and cellular homeostasis and bioenergetics. However, the shift of autophagy to lower or higher than physiological levels may give rise to cell death, by accumulation of toxic molecules and damaged organelles or by degrading many essential proteins and organelles, respectively.^7,37^ The role of autophagy in cardiac complications of diabetes is not well elucidated. Some studies demonstrated that during myocardial I/R injury the autophagic activity is enhanced to the detrimental level.^37^ There is also a controversy that whether modulating autophagy is protective or harmful for myocardial I/R injury.^7^ Recent experimental studies show that myocardial I/R injury and high fat diet lead to the upregulation of autophagy and impairment of autophagosome clearance.^38,39^ It has been demonstrated that autophagy flux in the heart under diabetes is impaired as shown indirectly by up-regulation of LC3B-II and p62 protein level^36,37^ or inhibited as shown by down-regulation of LC3B-II and up-regulation of p62.^38^ In our study, myocardial I/R injury led to the elevation of the expression of both proteins LC3B-II and p62 in diabetic hearts. Although the autophagic flux was not directly examined in the present study due to some limitations, related results of this experiment are consistent with previous studies, indicating the impairment of autophagy flux in diabetic and I/R hearts as compared with non-diabetic ones.

Mitochondrial ROS generation and resultant autophagosome accumulation are associated with further ROS overproduction and mitochondrial permeabilization, leading to cell death by apoptosis.^36^ Restoration of autophagosome clearance through adjusting the autophagic flux to an appropriate levels is thought to maintain cellular homeostasis and cell survival; this restoration was beneficial for the heart, by reducing cell apoptosis, oxidative stress and mitochondrial dysfunction in diabetic mice.^36,38^ Similarly, application of vildagliptin with or without IPostC reduced the expression levels of LC3B-II and p62, accompanying with improved mitochondrial function and cardioprotection. Even though IPostC alone reduced mitochondrial ROS generation considerably, this effect was not associated with a significant reduction in infarct size by this strategy. This may possibly because the other arms of protection process (e.g. restoration of p62 levels and autophagosome clearance) are not affected by IPostC during diabetes. This finding likely indicates that reduction of mitochondrial ROS by itself could not responsible for cardioprotection and there should be an effective co-activation or coordination of multiple protective mechanisms. However, further study could overcome our limitation in evaluation of the reliance of this hypothesis.

Beside its hypoglycemic effect, vildagliptin decreased mitochondrial ROS production and mitochondrial depolarization, increased mitochondrial counts and improved the indications of autophagy flux accompanied with significant cardioprotection in diabetic hearts with I/R injury. It is demonstrated that vildagliptin has various important effects including decreasing lipid profile,^40^ and anti-oxidative,^41^ anti-inflammatory,^42^ and anti-apoptotic^43^ features in type-II diabetes, which they can contribute partly to observed cardioprotective actions. Because of these benefits of vildagliptin, the myocardial salvaging effects and cardio–cerebrovascular safety of this DPP-4 inhibitor has been reported in animals and patients with type-II diabetes.^44^ In rats with isoproterenol-induced myocardial infarction (MI) and in non-diabetic obese rats with myocardial I/R injury, vildagliptin have reduced infarct size and recovered cardiac function through mitochondrial protection, and anti-oxidative and anti-apoptotic properties.^15,45^ In addition, in the OLETF, a rat model of type-II diabetes, vildagliptin reduced diabetes-induced post-MI acute mortality possibly by restoring the autophagic response through attenuation of Bcl-2-Beclin-1 interaction.^46^

Previous studies have shown that IPostC could not restore diabetic heart function and infarct size following I/R injury.^13,47^ Our studies proposed that IPostC could act more effectively if it was accompanied with a pharmacological agent.^13^ As mentioned for diabetes, there is no conclusive and unequivocal evidence about the exact effects of postconditioning on the autophagic activity following myocardial I/R injury. In some studies, for example, postconditioning with sevoflurane,^10^ and IPostC^9,48^ promote autophagic activity via inhibiting the phosphorylation of mTOR during early reperfusion in non-diabetic hearts. In contrast, other studies have shown that cardioprotective effect of IPostC was associated with the inhibition of autophagic activity by the regulation of beclin-1 and AMPK/mTOR signaling pathway^49^ or by preventing apoptotic and autophagic cell death in cardiomyocytes culture.^50^ Our present results were in accord with the latter, as we found IPostC alone or in combination with vildagliptin tended to reduce the levels of autophagic markers, LC3B-II and p62, in diabetic hearts. These diverse results may be related mostly to the designs of the basic and pre-clinical studies. IPostC is conducted with different algorithms, resulting in dissimilar effects in diabetic or healthy hearts. The important variables possibly leading to these differences are the variability in activation levels of cell survival pathways and mediators, and presence or absence of end effectors (e.g. normal mitochondrial function) in diabetic or healthy hearts, and intrinsic characteristics of the tissues as well as the methodological discrepancies.^51^ On the other hand, we observed that the mitochondrial and autophagy variables were affected constantly by combination of IPostC with vildagliptin and these effects were associated with full and greater cardioprotection rather than individual treatments. It seems that vildagliptin potentiates the cardioprotective influences of IPostC on diabetic myocardium. Due to the interfering effects of diabetes-induced changes with cardioprotection, it is plausible that the potency of IPostC may be reduced during diabetes. However, concomitant application of vildagliptin can normalize some abnormal metabolic and cellular changes of diabetes and thus increase the effectiveness of IPostC in this scenario, not in all but in most aspects of protective ways.

## Conclusion

Combination of vildagliptin and IPostC in type-II diabetic hearts was a well-working strategy to reduce myocardial I/R damages by restoring mitochondrial membrane potential and mitochondrial function and modulating the autophagic activity in risk zones of I/R hearts. However, additional studies are necessary to elucidate the contribution of signaling pathways in the regulatory effects of Vildagliptin/IPostC on autophagy and mitochondrial biogenesis and function, especially in the models of *in vivo* I/R injury.

## Acknowledgments

This work was supported in part by a grant from the National Elite Foundation of the Islamic Republic of Iran, and a grant from Drug Applied Research Centre, Tabriz University of Medical Sciences, Tabriz-Iran. The authors declare that they have no conflict of interest with respect to the research, authorship, and/or publication of this article.

## Ethical Issues

All steps of the experiment were carried out in line with the Guide for the Care and Use of Laboratory Animals published by the US National Institutes of Health (NIH publication No 85-23, revised 1996) and approved by the Ethical Commission of Tabriz University of Medical Sciences (ethical number: 5.4.10696).

## Conflict of Interest

The authors declare no conflict of interests.
